# Effect of national curriculum reform on medical students’ preparedness for practice: a prospective cohort study from undergraduate to postgraduate periods

**DOI:** 10.1186/s12909-022-03909-3

**Published:** 2022-11-30

**Authors:** Chung-Hsien Chaou, Shiuan-Ruey Yu, Shou-De Ma, Hsu-Min Tseng, Liang-Shiou Ou, Chien-Da Huang, Ji-Tseng Fang

**Affiliations:** 1 Department of Emergency Medicine, Chang Gung Memorial Hospital, Linkou, Taiwan; 2 Chang Gung University College of Medicine, Taoyuan, Taiwan; 3 Chang-Gung Medical Education Research Centre, Chang Gung Memorial Hospital, Taoyuan, Taiwan; 4 Tungs’ Taichung Memorial Hospital, Taichung, Taiwan; 5 Department of Health Care Management, Chang Gung University, Taoyuan, Taiwan; 6 Department of Pediatrics, Chang Gung Memorial Hospital, Taoyuan, Taiwan; 7 Department of Thoracic Medicine, Chang Gung Memorial Hospital, Taoyuan, Taiwan; 8 Department of Nephrology, Chang Gung Memorial Hospital, Taoyuan, Taiwan

**Keywords:** Preparedness for practice, Undergraduate medical education, Burnout, Longitudinal cohort study, Transition, Questionnaire, Mixed model

## Abstract

**Background:**

In recent years, a national curriculum reform was implemented in undergraduate medical education in Taiwan to reduce clinical rotation training from 3 years to 2 years. The last generation of the old curriculum and the first generation of the new curriculum both graduated in 2019. This study aimed to compare the learning outcomes of the medical students in these two curriculum groups in terms of preparedness for practice during the transition from undergraduate to postgraduate study.

**Methods:**

This was a 3-year prospective, longitudinal, comparative cohort study between 2017 and 2020. Medical students from both the 7-year and 6-year curriculum groups received biannual questionnaire surveys starting 18 months before graduation and running until 11 months after graduation. The measurement tools were the Preparedness for Hospital Practice Questionnaire (PHPQ) and Copenhagen Burnout Inventory (CBI). Personal demographic information was also collected. Linear mixed models were used to determine the effect of curriculum change on learners’ preparedness and burnout levels.

**Results:**

A total of 130 medical students from the two cohorts provided 563 measurements during the study period. Compared to their counterparts following the old curriculum, the participants following the new curriculum showed a lower level of preparedness when first entering clinical rotation (*p* = 0.027) and just after graduating (*p* = 0.049), especially in the domains of clinical confidence (*p* = 0.021) and patient management *p* = 0.015). The multivariate linear mixed model revealed gradual increases in preparedness and burnout in serial measurements in both curriculum groups. Students following the new curriculum, which involved a shortened clinical rotation, showed a slightly lower overall preparedness (*p* = 0.035) and the same level of burnout (*p* = 0.692) after adjustment. The factor of *year of change* did not show a significant effect on either preparedness (*p* = 0.258) or burnout (*p* = 0.457).

**Conclusion:**

Shortened clinical rotation training for medical undergraduates is associated with a decrease in preparedness for practice during the transition from undergraduate to postgraduate study. Clinical confidence and patient management are the main domains affected.

**Supplementary Information:**

The online version contains supplementary material available at 10.1186/s12909-022-03909-3.

## Introduction

Medical education is a continuing and life-long professional development process. The goal of such education is to build an adequate level of core competencies and prepare learners for independent clinical practice [1]. Curricula are created with prespecified educational outcomes, and corresponding learning content, teaching methods, assessment tools, and evaluation processes are designed [2, 3]. According to a survey by Wijnen-Meijer, the diverse medical education structure around the world can be described by six models and mainly three stages: the medical school stage, the postgraduate general practice (the ‘in between phase’), and the residency specialty training. Some countries accept medical students direct from secondary schools, and others require a bachelor’s degree. For postgraduate general practice, it may consist of postgraduate internship, mandatory service, both, or none [4]. From time to time, curriculum reforms are undertaken to improve learner experience, enhance learning outcomes, cultivate renewed target competencies, or adapt to altered national regulations [5–10]. Some reforms comprise major changes involving education systems for health care professionals [5, 6], while others may include changes only within individual schools, faculties, or even courses [10–13].

In 2012, a national curriculum reform was implemented in undergraduate medical education in Taiwan to reduce clinical rotation training from 3 years to 2 years. Traditionally, Taiwanese medical school education has consisted of a seven-year curriculum, including four years of premedical and basic medical programs and three years of clinical rotation learning courses [14]. The 3 years of clinical learning involve hospital-based, case-based learning and can be further divided into 2 years of observership (clerkship) and 1 final year of hands-on clinical placement (undergraduate internship). However, compared with the role of postgraduate internships in other countries, where they are conducted after graduation and board certifications, the role of nonlicensed interns in Taiwan may be confusing [1, 15, 16]. On the one hand, they are regarded as students since they have not yet graduated from medical school, but on the other hand, they are often seen as part of the workforce since they do provide some of the basic medical services such as charting, patient evaluation, and basic clinical tasks [17].

Efforts have been made in recent years to facilitate this curriculum change. For instance, traditional school-based and hospital-based learning have become more integrated, clinical courses and assessment methods have been discussed thoroughly among medical system educators, the competencies and clinical skills that a 6-year curriculum graduates should possess have been redefined, and PGY courses that are suitable for 6-year curriculum graduates have also been redesigned [17–20]. This study aimed to compare the learning outcomes of medical students following each of these curricula. However, it is rather difficult to compare two parallel curricula directly since their courses, clinical activities, assessments, and objective endpoints are independent of each other. Considering that one of the most important goals of medical education is to prepare learners for independent clinical practice, the preparedness for practice was conceptualized as both a long-term and a short-term venture that included personal readiness as well as knowledge, skills, and attitudes [21]. We evaluated the learners’ preparedness for independent hospital practice and level of burnout during the transition from undergraduate to postgraduate periods.

## Methods

### National curriculum reform

**Fig. 1:**
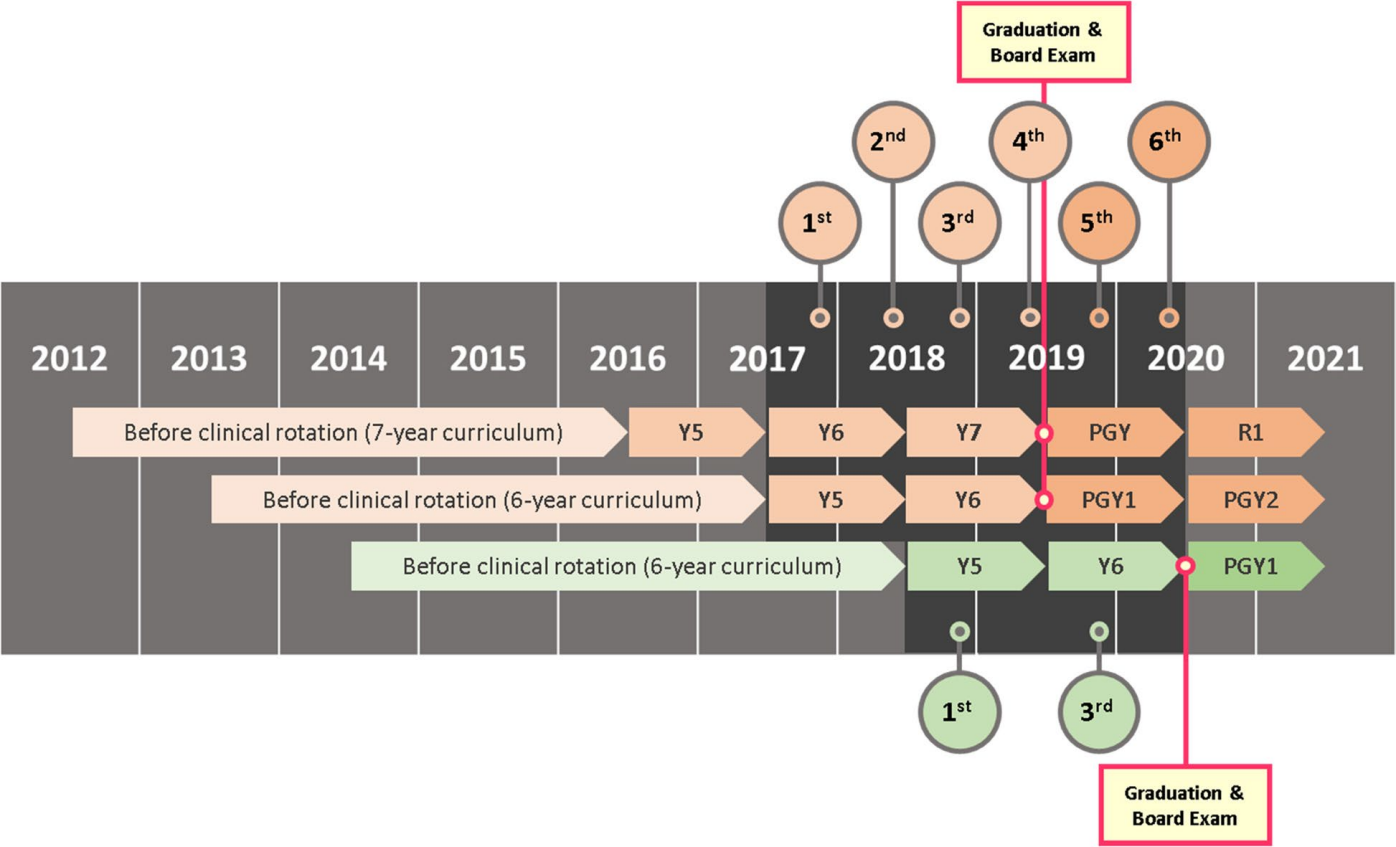
Diagram of the curriculum reform timeline. the enrolled cohort and the measurements are shown in chronological order

In 2000, the joint committee of deans of medical schools in Taiwan passed a curriculum reform resolution to improve the medical education system. An expert panel was formed to design the new 6-year medical system [18]. An experimental 6-year curriculum was tested at the National Taiwan University College of Medicine with selected students, and their learning results did not differ much from those of students following the original 7-year curriculum [15]. In 2009, the joint committee announced that a new medical education curriculum would be carried out in 2013, and the first year of the new curriculum would yield graduates in 2019. Following decreased undergraduate rotation, the postgraduate year (PGY) rotation was increased from 1 year to 2 years. PGY training is a general practitioner rotational program undertaken before further specialist training (residency) [14, 22]. After the outbreak of SARS (severe acute respiratory syndrome) in 2003, the Taiwan Ministry of Health and Welfare set up this one-year PGY training to improve medical graduates’ core competencies and prevent them from becoming specialized too early [17]. In a way, the reform placed the graduation and board certification 1 year earlier than they were previously (Fig. 1).

### Study setting

This was a prospective, longitudinal comparative cohort study conducted between 2017 and 2020. In July 2017, students of the last generation to receive the old curriculum (2012 cohort, 3 years of clinical rotation) and the first generation to receive the new curriculum (2013 cohort, 2 years of clinical rotation) started their last two years of clinical learning simultaneously. They spent their last two years in teaching hospitals completing their clinical rotation, graduated, took national board exams, and then started their postgraduate clinical learning at the same time (Fig. 1). A deidentified online survey was given to the participants at multiple prescheduled times. The study was approved by the Chang Gung Medical Foundation institutional review board (IRB No. 201601758B0, 201701981B0). All methods were carried out in accordance with relevant guidelines and regulations.

### Ethical approval

 The study was approved by the Chang Gung Medical Foundation institutional review board (IRB No. 201601758B0, 201701981B0).

### Participants

Undergraduate students admitted to Chang-Gung University College of Medicine in 2012 (old curriculum) and 2013 (new curriculum) were eligible to participate in the study. We invited them via the Internet to join this three-year longitudinal survey study from two years before graduation to one year after graduation. All participants were given a one-hour orientation before providing written informed consent. To eliminate the effect of *change* itself on learning experiences, we enrolled another group of participants from the 2014 cohort (new curriculum) using the same method described above. This group of participants followed the new curriculum but were enrolled one year after the *year of the change* and thus served as the control group among the new curriculum students.

During the first year of the study (2017/06-2018/05), the participants of the 2012 cohort and 2013 cohort entered their clinical rotation at the same teaching hospital (site A), which, with a 3600-bed capacity, is the largest teaching hospital in Taiwan. During the second year of the study (2017/06-2018/05), the participants of the 2014 cohort entered their clinical rotation in the same teaching hospital (site A), and the participants from the 2012 and 2013 cohorts were distributed among the four teaching hospitals in Taiwan (sites A, B, C, D) according to their preference. These four teaching hospitals, which differ in geographical location and patient population backgrounds, consist of two medical centers and two regional hospitals. The participants from both the 2012 and 2013 cohorts graduated in 2018/06 and took the national board exams in 2018/07. During the third year of the study (2018/06-2019/05), most of the participants from both the 2012 and 2013 cohorts began their mandatory postgraduate rotation (PGY) training. The PGY training lasts for one year in the old curriculum and two years in the new curriculum (Fig. 1). A few of the participants who did not proceed to PGY training either entered military service, in the case of some male graduates; prepared for another round of board exams due to failing their first attempt; or were lost during follow-up. A detailed list of the distribution of participants is provided in Supplementary file 1.

### Data collection and measurements

**Fig. 2:**
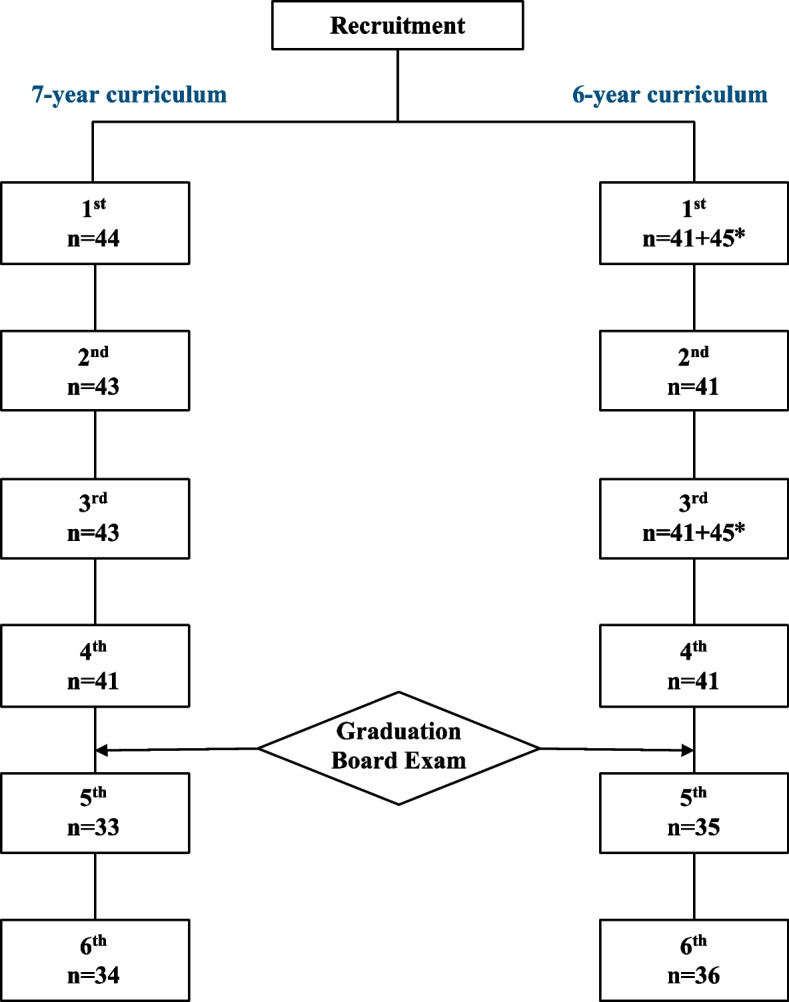
Diagram of serial measurements and number of respondents after recruitment. The number of participants from the 2014 cohort is marked with an asterisk

We collected basic personal demographic information including age, sex, and self-reported previous academic performance before entering clinical rotation. Learning-related variables included which study hospital they were assigned to. The participants from the 2012 and 2013 cohorts were asked to complete surveys regarding their preparedness and degree of burnout every 6 months after joining the study. These measures were labeled from 1 to 6 according to chronological order, with the 1^st^ to 6^th^ measurements being conducted at 18 months before graduation, 12 months before graduation, 6 months before graduation, 1 month before graduation, 5 months after graduation, and 11 months after graduation, respectively. The participants from the 2014 cohort, who served as a control group against the variable of *year of change*, completed only two surveys with a 1-year gap, which were spaced to match the timing of the 1^st^ and 3^rd^ measurements of the study group. A diagram of serial measurements and number of respondents in each measurement is presented in Fig. 2. The survey process was conducted online and anonymously, and respondents were instructed to answer the questions in reference to their rotations in the current and previous months.

Their preparedness for independent clinical practice was assessed using the Chinese version of the Preparedness for Hospital Practice Questionnaire (CPHPQ). This eight-domain measurement, consisting of 41 items evaluated on a six-point Likert scale, was previously developed and validated by Chaou et al. with good internal consistency (Cronbach's alpha=0.94) [23]. The eight subscale domains were interpersonal skills (IS), clinical confidence (CF), team collaboration (CL), patient management (MG), medical science (SC), disease prevention (PV), holistic care (HC), and self-directed learning (SDL).

We evaluated the degree of burnout using the Copenhagen Burnout Inventory (CBI), a measurement developed by Kristensen et al. to measure burnout in the populations of medical professionals [24]. The CBI measures three dimensions, including personal burnout, work-related burnout, and patient-related burnout, using a 5-point Likert scale. It has been shown to be more straightforward for measuring burnout among medical professionals than another frequently used questionnaire, the Maslach Burnout Inventory [25]. A Chinese version of the CBI has also been developed and validated in previous studies [26–28].

### Statistical analysis

We determined the sample size before the research was started. For the descriptive results, the mean and standard deviation (SD) are used to describe the central tendency and spread of continuous variables and the count and percentage for categorical variables. We used independent t tests for the comparison of continuous results between groups and a chi-square test or Fisher's exact test for categorical results, where appropriate. We treated the sum of the Likert scales as continuous variables in accordance with Norman et al. [29].

For the analysis of curriculum change on the preparedness and burnout of medical students during the graduation transition, we used linear mixed models to cope with dependence within the subject or training hospital while exploring the effects of possible explanatory variables [30]. Serial measurements of total PHPQ scores and CBI scores were taken as dependent variables. Individual learners and training sites were added into the model as random intercepts. The models tested the effect of curriculum change (old vs. new) and the year of change while adjusting for personal demographic variables, including sex, age, and self-reported previous academic performance. The models were fit using the residual maximum likelihood (REML) approach, and the covariance structure of the two random effects was set as unstructured. All analyses were performed using SAS statistical software version 9.3 (SAS Institute Inc., Cary, NC). A reported p-value of less than 0.05 was considered statistically significant.

## Results

A total of 130 medical students participated in this study, including 44 (40.4%) from the 2012 cohort (7-year curriculum), 41 (40.2%) from the 2013 cohort (6-year curriculum), and 45 (43.3%) from the 2014 cohort (6-year curriculum). A total of 563 measurements were taken during the 3-year period. Table 1 provides detailed personal demographic information and the number of students who participated in each survey from each group. Supplementary file 1 provides descriptions of the training sites and details on the distribution of participants over the three-year period.

**Table 1:** Demographics of the participants. Data are presented as numbers (percentages) unless stated otherwise

	Overall (*n* = 130)	7-year curriculum	6-year curriculum	
		**2012 cohort ** **(** ***n*** ** = 44)**	**2013 cohort ** **(** ***n*** ** = 41)**	**2014 cohort (** ***n*** ** = 45)**
Age at inclusion^*^	23.4 (1.39)	24.1 (0.91)	23.2 (1.42)	22.8 (1.48)
Male Gender	72 (55.4)	26 (59.1)	24 (58.5)	22 (48.9)
Geographic area				
North	69 (53.1)	20 (45.5)	20 (48.8)	29 (64.4)
Central	27 (20.8)	10 (22.7)	11 (26.8)	6 (13.3)
South	23 (17.7)	12 (27.3)	6 (14.6)	5 (11.1)
East	2 (1.50)	0 (0)	1 (2.40)	1 (2.20)
Offshore islands and overseas	9 (6.92)	2 (45.5)	3 (7.31)	4 (8.90)
Previous academic performance				
Top third	39 (30.0)	13 (29.5)	11 (26.8)	15 (33.3)
Middle third	62 (47.7)	20 (45.5)	21 (51.2)	21 (46.7)
Bottom third	29 (22.3)	11 (25.0)	9 (22.0)	9 (20.0)
Number of students participated				
1st measurement	130 (100)	44 (100)	41 (100)	45 (100)
2nd measurement	84 (98.8)	43 (97.7)	41 (100)	0 (0)
3rd measurement	129 (99.2)	43 (97.7)	41 (100)	45 (100)
4th measurement	82 (96.5)	41 (93.2)	41 (100)	0 (0)
5th measurement	68 (80.0)	33 (75.0)	35 (85.4)	0 (0)
6th measurement	70 (82.4)	34 (77.3)	36 (87.8)	0 (0)

* presented as mean (SD)

**Fig. 3:**
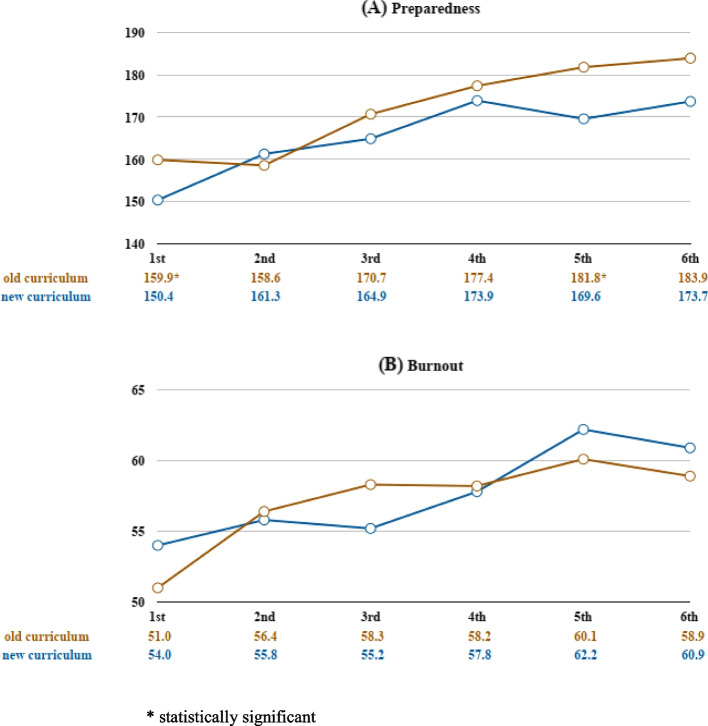
Comparison of serial measurement results between the two curriculum groups. The old curriculum is a 7-year curriculum with 3 years of clinical rotation. The new curriculum is a 6-year curriculum with 2 years of clinical rotation

The serial measurement results of preparedness and burnout are shown in Fig. 3. The results of the new curriculum included data from the 2013 and 2014 cohorts. A gradual increasing trend of both the level of preparedness and the level of burnout is seen from the 1^st^ to 6^th^ measurements. Compared to their counterparts, the participants following the new curriculum showed a significantly lower level of preparedness for practice when first entering clinical rotation (1^st^ measurement, 150.4 ± 23.3 vs. 159.9 ± 22.2, *p *= 0.027) and just after graduating and entering PGY training (5^th^ measurement, 169.6 ± 28.4 vs. 181.8 ± 20.9, *p* = 0.049). A comparison of the subscale domains of the PHPQ at the 5^th^ measurement, when the participants had just graduated and begun their independent practice, are listed in Table 2. The cohorts following the new curriculum reported a lower level of preparedness in the domains of clinical confidence (23.3 ± 4.93 vs. 25.8 ± 3.81, *p* = 0.021) and patient management (21.7 ± 3.56 vs. 23.7 ± 2.92,*p* = 0.015). Detailed total scores of each measure are provided in Supplementary file 2.

**Table 2:** Comparison of subscales of preparedness for hospital practice between to cohorts just after graduation (5th measurement). Data are presented as the mean (SD)

	Preparedness		
	Old curriculum	New curriculum	*p*-value
Interpersonal skills	14.4 (3.69)	13.6 (3.99)	0.3995
Clinical confidence	25.8 (3.81)	23.3 (4.93)	0.0213^*^
Team collaboration	17.6 (3.21)	16.6 (3.88)	0.2785
Patient Management	23.7 (2.92)	21.7 (3.56)	0.0148^*^
Medical Science	16.7 (2.57)	15.4 (3.51)	0.0886
Disease Prevention	29.3 (3.57)	27.7 (3.94)	0.0758
Holistic care	27.0 (3.65)	25.4 (4.53)	0.1214
Self-directed learning	27.3 (3.21)	25.9 (4.64)	0.1538

* statistically significant

The results of the multivariate linear mixed model are reported in Table 3. For the endpoint of preparedness for independent hospital practice, the second and subsequent measurements all showed increases in total PHPQ scores (2^nd^ measurement, *p* = 0.075; 3^rd^ to 6^th^ measurements, all *p* < 0.001), indicating a continual increase in averaged overall preparedness for hospital practice with time. Participants following the 6-year curriculum reported a significantly lower overall level of preparedness compared with their counterparts after adjustment (β=-9.98, *p* = 0.035). The factor of *year of change* did not show a significant effect on the total PHPQ score (*p* = 0.258), which means that the preparedness of the 2013 and 2014 cohorts did not show a significant difference. For the endpoint of burnout, similarly, the second (*p* = 0.01), fourth (*p* = 0.01), fifth (*p* = 0.001), and sixth (*p* = 0.01) measurements all showed a significant increase in total CBI scores. In addition, age was associated with a significant increase in the level of burnout (β=1.54 per 1-year increase in age, *p* = 0.01). The new curriculum (*p* = 0.692) and the year of change (*p* = 0.457) did not show a significant effect on the level of burnout.

**Table 3:** Result of the multivariate analysis of influential factors on learners’ preparedness and burnout

	Preparedness				Burnout			
	Crude		Adjusted		Crude		Adjusted	
	β	*p*-value	β	*p*-value	β	*p*-value	β	*p*-value
Age	7.73	< 0.001^a^	-1.54	0.2639	2.60	< 0.001^a^	1.54	0.0099^a^
Male gender	6.34	0.0866	6.08	0.0911	0.61	0.6925	-0.15	0.9240
Previous Academic Performance								
Top third	-0.08	0.9895	0.51	0.9173	-4.10	0.0567	-4.11	0.0520
Middle third	-0.06	0.9161	-2.59	0.5641	-1.72	0.3782	-2.06	0.2826
Bottom third	ref.	-	ref.	-	ref.		ref.	
Measurements								
1st	ref.	-	ref.	-	ref.		ref.	
2nd	4.10	0.0455^a^	3.64	0.0750	2.53	0.0055^a^	2.37	0.0100^a^
3rd	13.0	< 0.001^a^	14.3	< 0.001^a^	3.17	< 0.001^a^	1.63	0.0952
4th	19.8	< 0.001^a^	20.4	< 0.001^a^	4.46	< 0.001^a^	2.76	0.0121^a^
5th	20.7	< 0.001^a^	24.4	< 0.001^a^	8.29	< 0.001^a^	5.12	0.0018^a^
6th	22.7	< 0.001^a^	25.5	< 0.001^a^	7.25	< 0.001^a^	4.10	0.0114^a^
Curriculum								
Original	ref.		ref.		ref.		ref.	
New	-8.26	0.0305^a^	-9.98	0.0346^a^	-0.83	0.6052	0.79	0.6921
Year of Change								
No	ref.		ref.		ref.		ref.	
Yes	3.39	0.3855	5.06	0.2579	2.67	0.1003	1.42	0.4569

^a^Statistically significan

## Discussion

In this study, we compared the simultaneous learning experiences, in terms of preparedness for independent hospital practice and level of burnout, of two groups of medical students, each following a different curriculum from undergraduate to postgraduate study. The main findings were that a decrease in clinical rotation time results in medical students' decreased preparedness for hospital practice, especially during the transition period around graduation, and in the domains of clinical confidence and patient management.

The major changes between the two curricula were moving the 7^th^ year hands-on rotation to the postgraduate period and increasing PGY training from 1 year to 2 years. One result of this shortened clinical rotation, as shown from our results, was a decrease in the preparedness of medical graduates, especially in terms of patient management and clinical confidence. The implication of this is that the clinical skills and experience needed for patient management take time to develop. Good medical knowledge is not a substitute for experience with patient care. The above rationale is also supported by the slow but gradual increase in preparedness observed with each measurement. By immersing themselves in daily clinical patient care, the students' patient management abilities and clinical confidence grew. Similar results were found in the previous literature: medical graduates felt most unprepared in terms of technical skills and patient care, especially when managing complex clinical conditions or providing immediate care in medical emergencies [31–33]. Besides, trainees generally felt more confident and competent over time, and the most common reason for feeling unprepared was having insufficient prior "hands-on" experience [34]. In recent studies, empirical evidence also showed that there are individual and temporal differences regarding the progression of preparedness [23], and that challenging circumstances may negatively impact preparedness [21, 35].

The current study suggests that medical graduates following the new curriculum were not wholly unprepared but were generally less prepared than the students following the original curriculum. Indeed, it is generally considered that the clinical ability of graduates from the 6-year program has been somewhere between the clinical abilities of students in the sixth and seventh years in the original curriculum. Despite that the last year of undergraduate training is not completely removed from the system but rather moved to the postgraduate period, once the graduates are licensed, they are regarded as board-certified physicians and take full legal liability for their actions. The educators therefore must prepare learners to meet the expectations of society, their medical team, and even themselves upon graduation. Possible ways to fill this ability gap include integration of earlier PBL courses and targeting certain unprepared areas [36], novice boot camps [33], near-peer-assisted learning [37], or simulation-based training programs [38–40].

Burnout and emotional exhaustion during the initial clinical placement period have been widely documented, but most previous studies have been cross-sectional [41–44]. An interesting finding in this study is that the burnout level seems to increase as learners proceed further into their clinical rotations and gradually become more prepared. A possible explanation is that an observer or outsider experiences no burnout. As clinical placement is a gradual shift from observership to supervised hands-on learning, the more learners may engage in the clinical setting, the higher the level of burnout they experience may be. Another finding related to the level of burnout is that the older a learner is, the higher the level of burnout he or she reports. This could be explained by the fact that the intensive workload, transition from day to night shifts, change in environment and schedule differences between specialties all require considerable mental and physical energy. Some medical students who are several years older than their peers may experience a higher level of burnout. Further studies about the relationship between age and subsequent burnout during clinical placement are needed to address this issue.

### Limitations

This study has certain limitations. First, this study enrolled medical students from a single medical school. However, the study addressed medical students' clinical learning experiences, and differences in training hospitals were taken into account. Second, the study population consisted of mostly Taiwanese medical students, with only 5 foreign learners. The differences between cultural contexts must be considered before applying the results across settings. Third, the participants in this study were medical students who volunteered for the study. A selection bias may be present because students who participated may be different from the whole population in terms of their preparedness for practice or degree of burnout.

## Conclusion

A shortened duration of clinical rotatory training is associated with a decrease in preparedness for practice during the transition from undergraduate to postgraduate study. Clinical confidence and patient management were the main domains affected. Nevertheless, students from both curriculum groups showed a gradually increasing trend over the study period. Older learners were associated with an increased level of burnout during clinical rotation.

## Supplementary Information

**Additional file 1.**

## Data Availability

The datasets used and/or analysed during the current study available from the corresponding author on reasonable request.

## References

[1] DeZee KJ, Artino AR, Elnicki DM, Hemmer PA, Durning SJ (2012). Medical education in the United States of America. Med Teach.

[2] Harden RM (2000). The integration ladder: a tool for curriculum planning and evaluation. Med Educ.

[3] Harden R (2001). AMEE Guide No. 21: curriculum mapping: a tool for transparent and authentic teaching and learning. Med Teach.

[4] Wijnen-Meijer M, Burdick W, Alofs L, Burgers C, ten Cate O (2013). Stages and transitions in medical education around the world: clarifying structures and terminology. Med Teach.

[5] Xiao H, Xian L, Yu X, Wang J (2007). Medical curriculum reform in Sun Yat-sen University: implications from the results of GMER evaluation in China. Med Teach.

[6] Jablonover RS, Blackman DJ, Bass EB, Morrison G, Goroll AH (2000). Evaluation of a national curriculum reform effort for the medicine core clerkship. J Gen Intern Med.

[7] Figueiredo JF, Troncon LE, Rodrigues Mde L, Cianflone AR, Colares Mde F, Peres LC, Piccinato CE (2004). Effect of curriculum reform on graduating student performance. Med Teach.

[8] Bajow N, Djalali A, Ingrassia PL, Ragazzoni L, Ageely H, Bani I, Corte FD (2016). Evaluation of a new community-based curriculum in disaster medicine for undergraduates. BMC Med Educ.

[9] Coates WC, Crooks K, Slavin SJ, Guiton G, Wilkerson L (2008). Medical school curricular reform: fourth-year colleges improve access to career mentoring and overall satisfaction. Acad Med.

[10] Nie M, Gao ZY, Wu XY, Jiang CX, Du JH (2015). Evaluation of oral microbiology lab curriculum reform. BMC Med Educ.

[11] Lube MW, Borman KR, Fulbright AE, Friedell ML (2010). Retrospective evaluation of residents’ American Board of surgery In-Service training examination (ABSITE) scores as a tool to evaluate changes made in a basic science curriculum. J Surg Educ.

[12] Chowthi-Williams A, Curzio J, Lerman S (2016). Evaluation of how a curriculum change in nurse education was managed through the application of a business change management model: a qualitative case study. Nurse Educ Today.

[13] Frye AW, Carlo MD, Litwins SD, Karnath B, Stroup-Benham C, Lieberman SA (2002). Effect of Curriculum Reform on students’ preparedness for clinical clerkships: a comparison of three curricular approaches in one school. Acad Med.

[14] Chou J-Y, Chiu C-H, Lai E, Tsai D, Tzeng C-R (2012). Medical education in Taiwan. Med Teach.

[15] Liu KM, Lin CH (2011). The way of Medicine: the new six-year curriculum of the Department of Medicine. J Healthc Qual.

[16] Kozu T (2006). Medical education in Japan. Acad Med.

[17] Introduction to the historical context of the reform of the Taiwan Medical Department and curriculum integration planning https://www1.cgmh.org.tw/intr/intr2/ebmlink/36100/enews/me_epaper_106-04.htm.

[18] Tsai TC (2012). Planning and integration of the 6-Year curriculum in Medicine. J Healthc Qual.

[19] Liu M, Huang YS, Liu KM (2004). Minimal required clinical competencies of Medical Graduates-A study of competency oriented Medical Education. J Med Educ (Taiwan).

[20] Yang YM (2016). Educating Physicians for the 21st Century via the New Six-year Clinical Medical Curriculum planning the New Six-year Clinical Medical Curriculum in Taiwan based on the Experiences in the United States. J Healthc Qual.

[21] Monrouxe L, Bullock A, Cole JR, Gormley G, Kaufhold K, Kelly N, Mattick K, Rees CS, Scheffler G: How Prepared Are UK Medical Graduates for Practice? In. Edited by Council GM. UK; 2014.

[22] Chen CY (2009). The impacts of and methods of coping with the extension of the Post-Graduate Year General Medicine Training Program. J Med Educ (Taiwan).

[23] Yu SR, Cheng YC, Tseng HM, Chang YC, Ma SD, Huang CD, Hsieh MJ, Fang JT, Chaou CH. Undergraduates’ preparedness for practice is associated with professional identity and perception of educational environment: A validation study. Biomedical J. 2020. 10.1016/j.bj.2020.04.009. 10.1016/j.bj.2020.04.009 PMC8514794 34509426

[24] Kristensen TS, Borritz M, Villadsen E (2005). The Copenhagen Burnout Inventory: a new tool for the assessment of burnout. Work Stress.

[25] Winwood PC, Winefield AH (2004). Comparing two measures of burnout among dentists in Australia. Int J Stress Manage.

[26] Yeh WY, Cheng Y, Chen CJ, Hu PY, Kristensen TS (2007). Psychometric properties of the chinese version of Copenhagen burnout inventory among employees in two companies in Taiwan. Int J Behav Med.

[27] Cheng Y, Chen IS, Chen CJ, Burr H, Hasselhorn HM (2013). The influence of age on the distribution of self-rated health, burnout and their associations with psychosocial work conditions. J Psychosom Res.

[28] Chou LP, Li CY, Hu SC (2014). Job stress and burnout in hospital employees: comparisons of different medical professions in a regional hospital in Taiwan. BMJ Open.

[29] Norman G (2010). Likert scales, levels of measurement and the “laws” of statistics. Adv Health Sci Educ.

[30] Brown H, Prescott R (2015). Applied mixed models in Medicine.

[31] Mattick KL, Kaufhold K, Kelly N, Cole JA, Scheffler G, Rees CE, Bullock A, Gormley GJ, Monrouxe LV (2016). Implications of aligning full registration of doctors with medical school graduation: a qualitative study of stakeholder perspectives. BMJ Open.

[32] Monrouxe LV, Grundy L, Mann M, John Z, Panagoulas E, Bullock A, Mattick K (2017). How prepared are UK medical graduates for practice? A rapid review of the literature 2009–2014. BMJ Open.

[33] Minter RM, Amos KD, Bentz ML, Blair PG, Brandt C, D’Cunha J, Davis E, Delman KA, Deutsch ES, Divino C (2015). Transition to surgical residency: a multi-institutional study of perceived intern preparedness and the effect of a formal residency preparatory course in the fourth year of medical school. Acad Med.

[34] Daly M, Perkins D, Kumar K, Roberts C, Moore M (2013). What factors in rural and remote extended clinical placements may contribute to preparedness for practice from the perspective of students and clinicians?. Med Teach.

[35] Lundin RM, Bashir K, Bullock A, Kostov CE, Mattick KL, Rees CE, Monrouxe LV. “I’d been like freaking out the whole night”: exploring emotion regulation based on junior doctors’ narratives. Adv Health Sci Educ. 2018;23(1):7-28. 10.1007/s10459-017-9769-y PMC5801373 28315113

[36] Miles S, Kellett J, Leinster SJ (2017). Medical graduates’ preparedness to practice: a comparison of undergraduate medical school training. BMC Med Educ.

[37] Aba Alkhail B (2015). Near-peer-assisted learning (NPAL) in undergraduate medical students and their perception of having medical interns as their near peer teacher. Med Teach.

[38] Esterl RM, Henzi DL, Cohn SM (2006). Senior medical student “Boot Camp”: can result in increased self-confidence before starting surgery internships. Curr Surg.

[39] Fisher JW, Thompson BM, Garcia AD (2007). Integrative clinical experience: an innovative program to prepare for internship. Teach Learn Med.

[40] Laack TA, Newman JS, Goyal DG, Torsher LC (2010). A 1-week simulated internship course helps prepare medical students for transition to residency. Simul Healthc.

[41] Willcock SM, Daly MG, Tennant CC, Allard BJ (2004). Burnout and psychiatric morbidity in new medical graduates. Med J Aust.

[42] Lin CC, Lin BY, Lin CD (2016). Influence of clerks’ personality on their burnout in the clinical workplace: a longitudinal observation. BMC Med Educ.

[43] Sevencan F, Cayir E, Uner S (2010). Burnout status of interns and associated factors. Cah Sociol Demogr Med.

[44] Hannan E, Breslin N, Doherty E, McGreal M, Moneley D, Offiah G (2018). Burnout and stress amongst interns in irish hospitals: contributing factors and potential solutions. Ir J Med Sci.

