# Supplementary material for: Effect of national curriculum reform on medical students’ preparedness for practice: a prospective cohort study from undergraduate to postgraduate periods
Source: BMC Med Educ. 2022 Nov 30;22:826. doi: 10.1186/s12909-022-03909-3 (PMC9709741; doi:10.1186/s12909-022-03909-3)
Supplement: Supplementary file 1 — Additional file 1. [file 12909_2022_3909_MOESM1_ESM.docx]

**Supplementary material I.** Descriptive information of the training sites and the distribution of participants over the three-year period.

| Site | Description | location | Level of hospital | Capacity (beds) | 2012 (old  curriculum) | | |  | 2013 (new curriculum) | | |  | 2014 (new curriculum) | |
| --- | --- | --- | --- | --- | --- | --- | --- | --- | --- | --- | --- | --- | --- | --- |
|  |  |  |  |  | 2017 | 2018 | 2019 |  | 2017 | 2018 | 2019 |  | 2018 | 2019 |
| A | Teaching Hospital | Urban | Medical center | 3700 | 44 | 28 | 17 |  | 41 | 31 | 16 |  | 45 | 30 |
| B | Teaching Hospital | Rural | Regional hospital | 1089 |  | 4 | 4 |  |  | 4 | 2 |  |  | 3 |
| C | Teaching Hospital | Rural | Regional hospital | 1369 |  | 4 | 1 |  |  | 3 | 4 |  |  | 5 |
| D | Teaching Hospital | Urban | Medical center | 2686 |  | 8 | 1 |  |  | 3 | 1 |  |  | 7 |
| E | Teaching Hospital | Urban | Medical center | 2803 |  |  | 1 |  |  |  | 1 |  |  |  |
| F | Teaching Hospital | Urban | Medical center | 890 |  |  | 2 |  |  |  | 2 |  |  |  |
| G | Teaching Hospital | Urban | Medical center | 1383 |  |  | 1 |  |  |  | 0 |  |  |  |
| H | Teaching Hospital | Urban | Medical center | 1527 |  |  | 0 |  |  |  | 1 |  |  |  |
| I | Teaching Hospital | Rural | Medical center | 1244 |  |  | 1 |  |  |  | 0 |  |  |  |
| J | Military service | - | - | - |  |  | 5 |  |  |  | 4 |  |  |  |
| K | Failed board exam | - | - | - |  |  | 4 |  |  |  | 4 |  |  |  |
| L | Unknown | - | - | - |  |  | 7 |  |  |  | 6 |  |  |  |

**Supplementary file 2.**

Comparison of serial measurement results between the two curriculum groups. The score sums are presented as the mean (SD)

|  | Preparedness | | | | |  | Burnout | | | | |
| --- | --- | --- | --- | --- | --- | --- | --- | --- | --- | --- | --- |
|  | Old curriculum | | New curriculum | | p-value |  | Old curriculum | | New curriculum | | p-value |
| Measurements |  |  |  |  |  |  |  |  |  |  |  |
| 1^st^ (18 months before graduation) | 159.9 | (22.2) | 150.4 | (23.3) | 0.0274^*^ |  | 51.0 | (7.37) | 54.0 | (10.1) | 0.0516 |
| 2^nd^ (12 months before graduation) | 158.6 | (20.2) | 161.3 | (23.2) | 0.5731 |  | 56.4 | (10.3) | 55.8 | (8.22) | 0.7551 |
| 3^rd^ (6 months before graduation) | 170.7 | (22.2) | 164.9 | (22.1) | 0.1702 |  | 58.3 | (9.93) | 55.2 | (10.3) | 0.1125 |
| 4^th^ (1 month before graduation) | 177.4 | (22.3) | 173.9 | (26.8) | 0.5205 |  | 58.2 | (9.24) | 57.8 | (10.0) | 0.8281 |
| 5^th^ (5 months after graduation) | 181.8 | (20.9) | 169.6 | (28.4) | 0.0494^*^ |  | 60.1 | (9.83) | 62.2 | (11.4) | 0.4919 |
| 6^th^ (11 months after graduation) | 183.9 | (21.1) | 173.7 | (27.5) | 0.0888 |  | 58.9 | (12.5) | 60.9 | (10.4) | 0.5371 |

* statistically significant
